# CPEB3 Maintains Developmental Competence of the Oocyte

**DOI:** 10.3390/cells13100850

**Published:** 2024-05-16

**Authors:** Lucie Lamacova, Denisa Jansova, Zongliang Jiang, Michal Dvoran, Daria Aleshkina, Rajan Iyyappan, Anna Jindrova, Heng-Yu Fan, Yuxuan Jiao, Andrej Susor

**Affiliations:** 1 Laboratory of Biochemistry and Molecular Biology of Germ Cells, IAPG CAS, Rumburska 89, 277 21 Libechov, Czech Republic; 2 Department of Animal Sciences, Genetics Institute, University of Florida, Gainesville, FL 32610, USA; 3 Life Sciences Institute, Zhejiang University, Hangzhou 310058, China

**Keywords:** oocyte, embryo, translation, mRNA

## Abstract

Mammalian oocyte development depends on the temporally controlled translation of maternal transcripts, particularly in the coordination of meiotic and early embryonic development when transcription has ceased. The translation of mRNA is regulated by various RNA-binding proteins. We show that the absence of cytoplasmic polyadenylation element-binding protein 3 (CPEB3) negatively affects female reproductive fitness. CPEB3-depleted oocytes undergo meiosis normally but experience early embryonic arrest due to a disrupted transcriptome, leading to aberrant protein expression and the subsequent failure of embryonic transcription initiation. We found that CPEB3 stabilizes a subset of mRNAs with a significantly longer 3’UTR that is enriched in its distal region with cytoplasmic polyadenylation elements. Overall, our results suggest that CPEB3 is an important maternal factor that regulates the stability and translation of a subclass of mRNAs that are essential for the initiation of embryonic transcription and thus for embryonic development.

## 1. Introduction

The decline in female fertility is often attributed to poor oocyte quality, characterized by growth defects, incomplete meiotic divisions, and the inability to support embryonic development [1]. The underlying causes are mostly unknown, but are often associated with the disturbances in maternal factors [2,3,4,5]. The growing oocyte is transcriptionally active and accumulates large amounts of mRNA to meet the substantial proteomic demand during a long period of transcriptional quiescence between maturation and the 2cell stage in mice and the 8cell stage in humans, when the embryonic genome is fully reactivated [6,7]. However, the meiotic events and the transition from oocyte to embryo require a temporally restricted production of the proteins that control cell cycle progression and influence epigenetic reprogramming and cell fate determination during early development [8,9]. Therefore, the precise post-transcriptional regulation of maternally stored mRNAs has evolved to coordinate their translation and stability in a spatio-temporal manner [4,10,11,12]. The fate of a given mRNA is encoded in its own structure by specific sequence motifs located in the 3’ and 5’ untranslated regions (UTRs) that provide binding sites for RNA-binding proteins. The RNA–protein complex formed can increase/decrease the affinity of the mRNA for the translation initiation machinery, recruit degradation enzymes, or localize the mRNA [13,14,15,16].

The 3’-poly(A) tail plays a critical role in regulating both mRNA translation and turnover [16,17]. PolyA-binding proteins facilitate cap-dependent translation by bridging the 3′- and 5′-UTRs through interaction with translation initiation factors. Cytoplasmic polyadenylation (CPA) involves elongation of the 3’ poly(A) tail post nuclear polyadenylation and export and provides a mechanism for the recruitment of quiescent mRNA for translation in successive waves during oocyte and embryonic development and in somatic cells [18,19,20,21].

CPA is mainly regulated by canonical CPE elements (UUUAU) and non-canonical U-rich elements at the 3′UTR [22,23], which serve as binding sites for cytoplasmic polyadenylation element-binding proteins (CPEBs). In the basal state, CPEB acts as a repressor by attracting ribonuclease, which competes with cytoplasmic poly(A) polymerase bound to PAS via the CPSF complex [19,24,25]. As first shown in Xenopus oocytes [24], activation by upstream kinases after meiotic resumption promotes CPEB release and transcript polyadenylation [26,27]. There are four members of the CPEB family in vertebrate genomes, all of which are highly expressed in reproductive organs [28,29]. In oocytes, CPEB-dependent polyadenylation via CPEB1 is essential for meiotic progression: CPEB1 accumulates in fully grown GV and, activated by ERK1/2 phosphorylation, promotes translation of key components of the cell cycle [18,30]. Its degradation in metaphase I is required for the transition to MII in both *Xenopus* and mammalian oocytes [27,31,32]. CPEB2, studied in porcine oocytes, shows a similar pattern [33].

However, there is evidence that many important mRNAs are recruited for translation later in the course of meiosis and accumulate in MII, suggesting that mechanisms other than those dependent on CPEB1 must be involved [34,35]. In Xenopus oocytes, CPEB4 has been identified as the protein that replaces CPEB1 and regulates the cytostatic factors responsible for meiotic progression between MI and MII [36]. However, neither meiosis nor fertility were impaired in CPEB4-null mouse models [31,37], suggesting that there must be a different mechanism in mammals.

The last member of the family, CPEB3, is being studied in depth due to its link to synaptic plasticity in neuronal tissues. Despite the high expression of CPEB3 in reproductive organs, there are few studies on CPEB3 in reproduction, mostly on somatic cells [31,38,39,40], and direct evidence that germline-expressed CPEB3 affects female fertility is still lacking. CPEB3 is upregulated during maturation in mouse oocytes and zygotes [41,42].

To investigate the molecular role of CPEB3 in oocyte and early embryonic development, we generated a conditional knockout mouse strain. We found that the maternal polyadenylation factor CPEB3 affects female fertility by influencing early embryonic development through stabilization and translation of a subset of maternal transcripts in oocytes.

## 2. Material and Methods

### 2.1. Oocyte and Embryo Isolation and Cultivation

Six–ten-week-old BL6 females were superovulated by the intraperitoneal injection of 5 IU of pregnant mare serum gonadotropin (PMSG). GV oocytes were obtained from dissected ovaries 46 h after stimulation. Collected oocytes were handled in pre-warmed M2 transfer medium (Germany, Darmstadt, Merck) supplemented with 100 μM of IBMX (3-isobutyl-1-methylxanthine, phosphodiesterase inhibitor; Germany, Osterode, Sigma) to prevent nuclear envelope breakdown (NEBD). For in vitro maturation, selected oocytes were denuded, washed twice in transfer medium [43], and cultured in M16 medium (Germany, Darmstadt, Millipore) without IBMX at 37 °C, 5% CO_2_ for another 13–16 h. After 70 min, the oocytes that had progressed through NEBD were selected. To obtain MII oocytes in vivo, 5 IU hCG (Israel, Rehovot, ProSpec) was administered 48 h post PMSG. Zygotes were obtained from PMSG-primed females mated with males 17 h post hCG and cultured in vitro in 20 µL M16 medium under paraffin oil (Ovoil; Sweden, Vitrolife). 2cell embryos were collected 37 h post hCG administration. For the studies on subfertility, the zygotes were cultivated up to the blastocyst stage and the development rates were determined. For further processing, the samples were washed (3×) in a 0.1% solution of polyvinyl alcohol (PVA; Sigma) in phosphate-buffered saline (PBS) and frozen to −80 °C. Recombinant CPEB3 protein (H00022849-P01; Czech Republic, Prague, Bio-Techne) was injected into the GV oocyte at a final concentration of 40 ng/µL using an Eppendorf microinjection system. The PBS-injected CPEB3^+/+^ oocytes were used as controls. *CPEB3^LoxP/−^*; *ZP3^Cre+/−^* or CPEB3^+/+^ represents the WT oocyte/genotype, and *CPEB3^LoxP/LoxP^*; *ZP3^Cre+/−^* represents the cKO or CPEB3^−/−^ oocyte/genotype. All animal work was conducted according to Act No. 246/1992 on the protection of animals against cruelty.

### 2.2. Library Preparation and RNA-Sequencing

For total transcriptome analysis, RNA was isolated from five cells in quadruplicates using Trizol reagent (Merck) and dissolved in RNAse-free water. Quality assessment of the samples was performed with an RNA 6000 Pico Chip (USA, Santa Clara, CA, Agilent Technologies) using a Bioanalyzer instrument (Agilent). The RNA-seq libraries were generated from individual fractions by using the Smart-seq2 v4 kit (USA, Palo Alto, CA, Clontech) with minor modifications of the manufacturer’s instructions. Briefly, individual cells were lysed, and mRNA was captured and amplified with the Smart-seq2 v4 kit. After AMPure XP beads (Czech Republic, Beckman) purification, the amplified RNAs were quality checked by using the High Sensitivity D5000 kit (USA, Agilent Technologies). High-quality amplified RNAs were subject to library preparation (Nextera XT DNA Library Preparation Kit; USA, Illumina) and multiplexed by Nextera XT Indexes (USA, San Diego, CA, Illumina). The concentration of sequencing libraries was determined by using the Qubit dsDNA HS Assay Kit (USA, Waltham, MA, Life Technologies) and KAPA Library Quantification Kits (USA, Waltham, MA, KAPA Biosystems). The size of the sequencing libraries was determined by the High Sensitivity D5000 Assay in a TapeStation 4200 system (Agilent). Pooled indexed libraries were then sequenced on the Illumina HiSeq X platform with 150 bp paired-end reads. The acquired reads were trimmed using Trim Galore v0.4.1 and mapped onto the mouse reference genome assembly GRCm38 using Hisat2 v2.0.RNA expression was quantified as fragments per kilobase per million (FPKM) values in Seqmonk v1.40.Polysome-bound RNA data were obtained via SSP-Profiling [44] and presented in Iyyappan et al. (2023) [45]. The global transcriptome data were submitted to NCBI: GSE239545 at: https://www.ncbi.nlm.nih.gov/geo/query/acc.cgi?acc=GSE239545 (accessed on 28 July 2023).

### 2.3. RNA Isolation and cDNA Synthesis

RNA was isolated from 10 oocytes/embryos at the indicated stages. To avoid the loss of scarce material, the entire procedure was performed in a single tube without any sample transfer, using a TATAA CelluLyser Micro Lysis and DNA Synthesis Kit. The cells were lysed in 5 µL of CelluLyser™ buffer added directly to the sample tubes and kept on ice for 10 min to perform lysis. cDNA synthesis was carried out by RT-PCR using both oligo-dT primers and random hexamers with the following conditions: 5 min at 22 °C, 30 min at 42 °C, and 5 min at 85 °C for enzyme inactivation. To exclude the potential bias caused by the single-tube RNA isolation, we performed simultaneous RNA isolation by spin column extraction with an RNeasy Plus Micro Kit (Germany, Qiagen). cDNA from column-isolated RNA was synthesized with a qPCRbio cDNA synthesis kit (UK, London, PCR Biosystems). The obtained results showed no differences in transcript levels between the two methodological approaches.

### 2.4. PCR and qPCR

Real-time quantitative PCR (qPCR) assays were performed using QuantStudio 3^®^ (Netherlands, Bleiswijk, Applied Biosystems). To perform qPCR, Luna^®^ Universal qPCR master mix (USA, Ipswich, MA, New England Biolabs) was used according to the manufacturer’s instructions, supplemented with sample cDNA and 0.5 µL of the forward and reverse primers (Supplementary Table S1). Each qPCR run was performed in technical duplicates. The mRNA levels were normalized to the *Gapdh* reference gene, using the ΔΔ*Ct* calculation method CIT for relative quantification. General PCR experiments for the purpose of primer validation and mouse genotyping were conducted with PPP master mix (Czech Republic, Praha, TOP-Bio), under the following PCR conditions: 1 min at 94 °C, 18 secs at 94 °C, 18 secs at 58 °C, and 15 secs at 72 °C. The products were separated on 1.2% agarose gel with GelRed (41003; USA, Fremont, CA, Biotinum) and developed in an Azure 600 imaging system (USA, Dublin, CA, Azure Biosystems).

### 2.5. PolyA Tail Length Assay

The experimental procedure was based on the protocol by Sallés and Strickland (1999) [46], with the following modifications. To keep the entire length of the polyA tail of each transcript, the total RNA was extracted by the phenol-chloroform method. Isolated RNA was incubated with 1 µL of 50 μM oligo-dT per sample for 5 min at 65 °C. The ligation mix was prepared from the following components: T4 ligase (1 µL), Superscript IV 5× buffer (5 µL), 20U/µL RNAse inhibitor (1 µL), 10 mM dNTP (1 µL), 10mM ATP (1 µL), 1M MgCl2 (0.1 µL), 0,1M DTT (2 µL), and RNAse-free water (2 µL). Each sample was processed in 10 µL of the mixture and incubated for 30 min at 42 °C to allow T4-mediated oligo-dT ligation. Anchoring was carried out by incubating with 1 µL of oligo-dT anchor (*5′-GCGAGCTCCGCGGCCGCGT-3′*) for 1 h at 12 °C, followed by 2 min of incubation at 42 °C. cDNA synthesis was performed by the addition of 1 µL of Superscript II Reverse Transcriptase with the following setup: 45 min at 45 °C, 10 min at 80 °C, hold at 4 °C. Prepared cDNA was subjected to PCR with gene-specific forward primers and anchoring reverse primer (Supplementary Table S1). Images were developed in an Azure 600 (Azure Biosystems). Each experiment was carried out in technical triplicates.

### 2.6. Transcription Assay

The 5-EU was added to the M16 medium and incubated with zygotes overnight. The resulting 2cell embryos were fixed in 4% paraformaldehyde/PBS for 15 min, permeabilized with 0.1% Triton X-100/PBS for 10 min at room temperature, and incubated with the Click-iT reaction cocktail for 1 h at room temperature in the dark. After incubation, the oocytes were washed once with the PBS and mounted onto slides with Vectashield (H-1500; USA, Burlingame, CA, Vector laboratories). The images were scanned in a Leica SP5 confocal laser-scanning microscope (Leica Microsystems, Wetzlar, Germany).

### 2.7. Western Blotting

The lysed oocytes or embryos were subjected to 4–12% SDS–polyacrylamide gel electrophoresis. The samples were transferred to a polyvinylidene difluoride membrane (Immobilon P; Germany, Merck Millipore) using a blotting system (Germany, Göttingen, Biometra GmbH) at 5 mA/cm^2^ over 25 min. The membranes were blocked for 1 h at room temperature and incubated at 4 °C overnight with the primary antibodies (Supplementary Table S1) with 1% milk/TTBS (Tween-Tris-buffer saline; NaCl, Tween 20, 2M; Tris pH 7,6; dH_2_O). After 3 cycles of 10 min of washing in 0.05% TTBS, the membrane was incubated at room temperature for 1 h in 3% milk with a secondary antibody conjugated with peroxidase (USA, West Grove, PA, Jackson Immunoresearch). After the washing step with 0.05% TTBS, the proteins were visualized by chemiluminescence (UK, Hatfield, Amersham, GE Healthecare Life Science) according to the manufacturer’s instructions.

### 2.8. Measurement of Overall Protein Synthesis

To measure the overall protein synthesis, 50 μCi of ^35^S-methionine [47] (Czech Republic, Pernštýnská, Perkin Elmer) was added to methionine-free culture medium for 12 h. Five oocytes or embryos per sample were lysed in SDS-buffer and subjected to SDS–polyacrylamide gel electrophoresis. Radioactively labelled proteins were detected by image plates and were scanned using BasReader (Japan, Tokyo, FujiFilm). Signal quantification was performed with the software ImageJ/FiJi 2.9.Next, the membranes were probed with a GAPDH antibody as a loading control.

### 2.9. Motive Analysis

The 3’UTR sequences of each group were mapped against the GRCm39 mouse reference genome (http://genome.ucsc.edu/cgi-bin/hgTables (accessed on 28 July 2023)). The 3′ UTR length and motif number were calculated with the R package Biostring (version 2.40.2) (the PAS motifs include “UUUUAU”, “UUUUAAAU”, “UUUUAAGU”, “UUUUACU”, and “UUUUCAU”, and the CPE motifs include “AAUAA” and “AUUAAA”). The violin diagrams were plotted with https://www.bioinformatics.com.cn (accessed on 28 July 2023), a free online platform for data analysis and visualization. The relative positions of the motifs in the transcripts and the corresponding distribution maps were calculated and plotted with the software MS Excel (version 2016, Microsoft). The total motif number at each position was plotted with OriginPro8.5.

### 2.10. Statistical Analysis

Mean and standard error values were calculated in MS Excel. The Student’s *t*-test was conducted in the software GraphPad 5 (USA, Washington DC, Prism) to determine statistical significance between groups. * *p* < 0.05 was considered to be statistically significant (labelled with an asterisk). ** *p* < 0.01 and *** *p* < 0.001.

## 3. Results

### 3.1. CPEB3 Is Actively Translated during Oocyte Maturation

**Figure 1:**
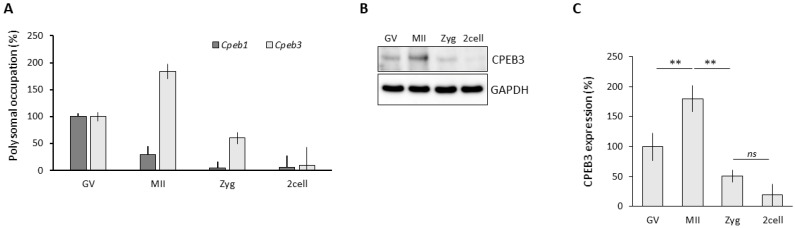
CPEB3 is actively translated during oocyte maturation. (**A**) Quantification of polysomal occupation of *Cpeb1* and *Cpeb3* mRNAs in oocyte and early embryo. (**B**) CPEB3 protein expression in oocyte and early embryo. The representative images are from three biological replicates. The values from GV were set to 100%. (**C**) Quantification of CPEB3 protein expression. Data are represented as the mean ± SEM of at least three independent experiments; ns, not significant; ** *p* < 0.01 according to one-way ANOVA. The values from GV were set to 100%.

To analyze CPEB3 translation in the mouse oocyte and early embryo, we performed polysomal fractionation, which showed that CPEB3 had a comparatively higher polysomal abundance at the MII oocyte stage relative to the CPEB family member CPEB1, which is active in the GV oocyte [27,48]. This observation indicated a higher CPEB3 translation (Figure 1A). Further CPEB3 immunoblotting analysis revealed a significantly higher CPEB3 expression in the MII oocyte, which gradually decreased as the 2cell stage of the embryo approached (Figure 1B,C).

Our data demonstrated that CPEB3 is actively translated and expressed in the MII oocyte.

### 3.2. Significantly Decreased CPEB3 in the Oocytes, Leading to Subfertility

**Figure 2:**
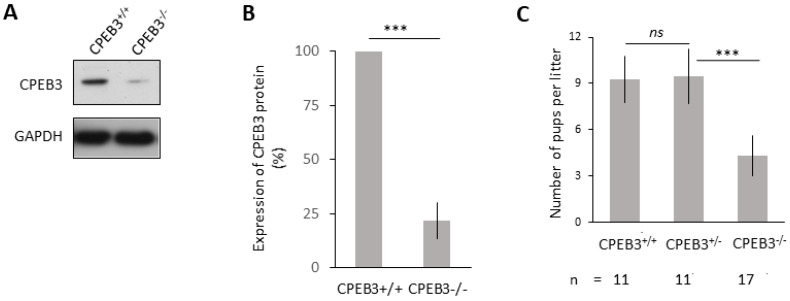
Significantly decreased CPEB3 in the oocytes, leading to subfertility. (**A**) CPEB3 protein expression in wild-type (CPEB3^+/+^ and cKO (CPEB3^−/−^) oocytes. The representative images are from six biological replicates. For CPEB3 mRNA expression, see Figure S1. (**B**) Quantification of CPEB3 protein expression in (**A**). Data are represented as the mean ± SEM of six independent experiments; *** *p* < 0.001 according to one-way ANOVA. The values from CPEB3^+/+^ were set to 100%. GAPDH was used as a loading control. (**C**) Quantification of number of pups per litter for females with specific genotype. Data are represented as the mean ± SEM; the number of breeding pairs is depicted below; ns, non-significant; *** *p* < 0.001 according to one-way ANOVA. For additional analyses of reproductive fitness, see Figure S1.

To perform more detailed analyses of the CPEB3 function, we generated oocyte-specific conditional knockout female mice (cKO), combining the CreLox system with the ZP3 promoter (see the Methods section). cKO oocytes (*CPEB3^LoxP/LoxP^; ZP3^Cre+/−^*, genotype, hereafter CPEB3^−/−^) exhibited the absence of *Cpeb3* mRNA (Figure S1A). The following immunoblot analysis showed a significant reduction in CPEB3 protein levels in the oocytes from the homozygous cKO females (CPEB3^−/−^, Figure 2A,B). To test the hypothesis that maternal CPEB3 is an important factor for female fertility, homozygous and heterozygous cKO females were mated with wild-type males. Significantly reduced litter size was observed in the homozygous CPEB3^−/−^ mice, compared to the wild-type (Cpeb3^+/+^). However, the presence of a single wild-type allele (CPEB3^+/−^) did not lead to a reduction in litter size (Figure 2C), indicating that there is no CPEB3 haploinsufficiency in the oocyte and the resulting zygote.

Here, we generated an experimental model for the CPEB3 deficiency that leads to female subfertility.

### 3.3. Oocytes with Downregulated CPEB3 Are Not Able to Sustain Preimplantation Embryo Development

Initially, to explore the cause of compromised CPEB3 cKO female fertility in more detail, we assessed folliculogenesis and oocyte quality. The ovaries from CPEB3^+/+^ and CPEB3^−/−^ mice were dissected and analyzed for their size, shape, and follicle density. The results did not show significant differences in ovarian morphology between genotypes (Figure S1B), with no differences between genotypes in mating (Figure S1C).

**Figure 3:**
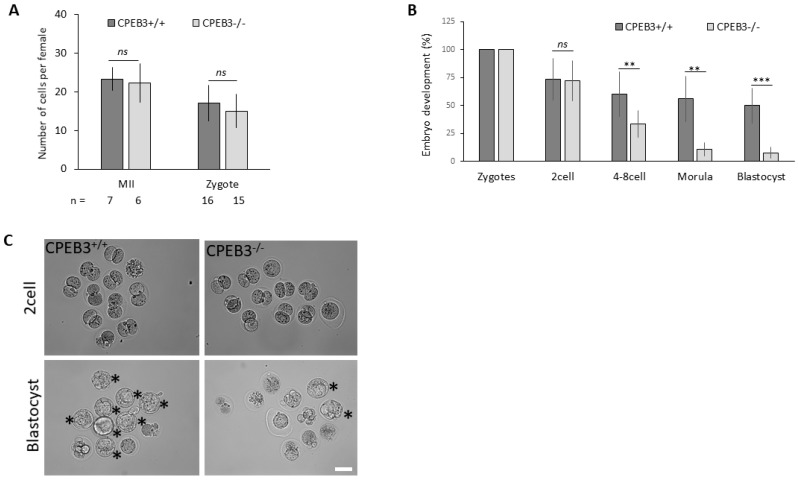
Oocytes with downregulated CPEB3 are unable to sustain preimplantation embryo development. (**A**) Quantification of oocyte morphology and fertilization. Data are represented as the mean ± SEM; the number of independent experiments is depicted below; ns, non-significant according to one-way ANOVA. (**B**) Quantification of post-fertilization embryo development using natural mating. Data are represented as the mean ± SEM from at least eleven independent experiments; ns, non-significant; ** *p* < 0.01; *** *p* < 0.001 according to one-way ANOVA. The values from zygotes were set to 100%. (**C**) Representative images of embryo development. Asterisks depict normally developed blastocysts. Scale bar, 100 µm. For the morphology of ovaries, see Figure S1.

Next, suggesting the role of CPEB3 in late meiosis, we isolated ovulated MII oocytes and zygotes from CPEB3^+/+^ and CPEB3^−/−^ mice. Unexpectedly, both genotypes produced a similar quantity and quality of collected oocytes in vivo as well as fertilized zygotes (Figure 3A and Figure S2), indicating that CPEB3 is dispensable for both folliculogenesis and meiotic progress to metaphase II, as well as fertilization.

To identify the underlying cause of the aberrant fertility phenotype in meiotic fully competent cKO oocytes, the experiments continued to investigate the subsequent embryonic development. To ensure the impact of maternally derived CPEB3 deficiency, cKO females were mated with WT males. The isolated zygotes showed a similar number and quality of fertilized zygotes derived from CPEB3^−/−^ oocytes. Interestingly, prolonged in vitro zygote cultivation revealed a significant reduction in developmental potential beyond the 2cell stage (Figure 3B). Moreover, a very low number of embryos reached the blastocyst stage (Figure 3B,C).

Overall, by utilizing breeding experiments together with the examination of isolated oocytes and zygotes, we demonstrated that the impaired female fertility in our experimental model for CPEB3 deficiency was caused by a developmental incompetency of CPEB3^−/−^ oocytes.

### 3.4. CPEB3 Depletion Results in Decreased Global Protein Synthesis and Transcriptional Activity of the 2cell Embryo

**Figure 4:**
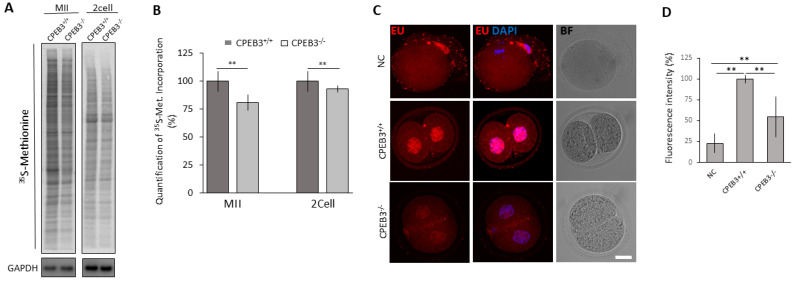
Depletion of CPEB3 leads to reduced global protein synthesis and transcriptional activity in the 2cell embryo. (**A**) Visualization of 35S-methionine incorporation into nascent synthesized proteins. *n* ≥ 4 of the biological replicates, GAPDH was used as loading control. (**B**) Quantification of visualization of 35S-methionine incorporation. Data are presented as mean ± SEM of at least four independent experiments; ** *p* < 0.01 according to one-way ANOVA. (**C**) Fluorescent labelling of newly transcribed RNA with 5-ethynyluridine (EU, red) in 2cell embryo. Transcriptionally inactive MII oocytes were used as negative control (NC); *n* ≥ 30, DNA blue; BF, bright field. For analysis of embryo cleavage timing, see Figure S2. (**D**) Quantification of EU fluorescence intensity. Data are presented as mean ± SEM; values of CPEB3^+/+^ embryos were set as 100%; ** *p* < 0.01 according to one-way ANOVA.

CPEBs are involved in mRNA metabolism and translation. To investigate the effect of its deficiency in oocytes and embryos, we analyzed the ^35^S-methionine uptake, which serves as a marker of translational activity. CPEB3 cKO MII oocytes and derived 2cell embryos have a slightly but significantly decreased global translational rate (Figure 4A,B). Further transcriptional activity analysis in the 2cell embryo by 5-ethynyl uridine (EU) labelling showed a significant decrease in the embryos derived from CPEB3^−/−^ oocytes (Figure 4C,D). Importantly, the timing to 2cell cleavage was similar between groups (Figure S2B).

**Figure 5:**
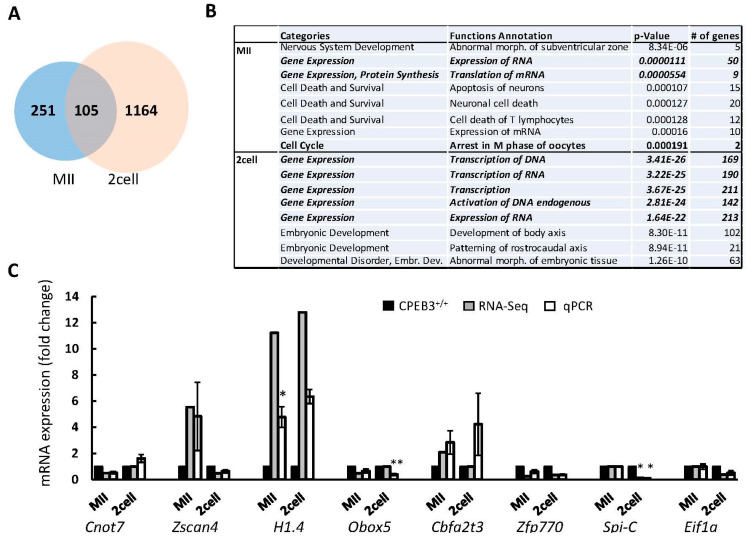
Absence of CPEB3 affects specific mRNAs in the oocyte and embryo. (**A**) Venn diagram shows number of differentially expressed RNAs, detected by RNA sequencing. Intersection depicts overlapping genes between two stages. Also see Supplementary File S1. (**B**) Top 8 GO cluster enrichment of genes that are differentially expressed in CPEB3^−/−^ MII oocytes and 2cell embryos compared to CPEB3^+/−^. Also see Supplementary File S1. (**C**) Validation of candidate genes from RNA-sequencing datasets (grey columns) by qPCR (white columns). Data are represented as the mean ± SEM; the value from CPEB3^+/−^ was set as 1; * *p* < 0.05, ** *p* < 0.01 according to one-way ANOVA.

Next, to assess the transcriptomes of CPEB3^+/−^ and CPEB3^−/−^ oocytes and their consequent 2cell embryos, we performed RNA sequencing (Figure S3). The acquired datasets showed significantly differentially expressed (DE) transcripts, with more than 1.5-fold difference in 356 mRNAs in the MII CPEB3^−/−^ oocyte and 1269 mRNAs in the 2cell embryo derived from the CPEB3^−/−^ oocyte (Figure 5A). Gene ontology analysis showed that DE mRNAs code mostly for the factors involved in RNA expression, translation, and transcription (Figure 5B and Supplementary File S1) in both oocytes and embryos. We selected candidate transcripts from the DE mRNA datasets related to the decreased developmental competence of MII CPEB3^−/−^ oocytes and the resulting 2cell embryos: *Cnot7*, *Zscan4*, *Histone 1.4*, *Obox5*, *Cbfa2t3*, *Zfp770*, *Spi-C*, and *Eif1a*. The subsequent validation of the candidate mRNAs by qPCR showed consistency with the RNA-seq datasets (Figure 5C).

### 3.5. CPEB3 Absence Influences Translation of Specific mRNAs via Polyadenylation

**Figure 6:**
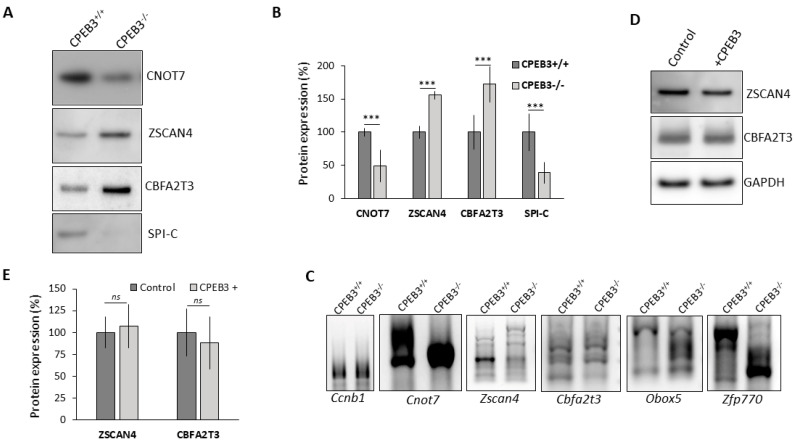
Absence of CPEB3 influences translation of specific mRNAs via polyadenylation. (**A**) Immunoblot analysis of expression of candidate proteins in MII oocytes (CNOT7, ZSCAN4, CBFA2T3) and embryos (SPI-C). The representative images are from at least three biological replicates. (**B**) Quantification of protein expression. Data are represented as the mean ± SEM of at least three independent experiments; *** *p* < 0.001 according to one-way ANOVA. (**C**) Polyadenylation assay (PAT) of candidate proteins in MII. Cyclin B1 mRNA (Ccnb1) was used as a negative control. The representative images are from at least three biological replicates. (**D**) Microinjection of CPEB3 protein into CPEB3^−/−^ GV oocytes normalizes specific protein expression similarly to that in the CPEB3^+/+^ MII oocyte (control). Representative images from at least three biological replicates. For analysis of CPEB3 expression see Figure S4. (**E**) Quantification of the protein expression from (**D**). Data are represented as the mean ± SEM of at least three independent experiments; ns, non-significant according to one-way ANOVA. CPEB3^+/+^ MII oocytes were used as a control and set as 100%.

Our previous results showed that the absence of CPEB3 in MII oocytes leads to decreased global translation (Figure 4A,B) and destabilization of particular transcripts within the oocyte (Figure 5). Furthermore, by immunoblotting we confirmed the protein expression of RNA-seq candidate mRNAs that were up- or downregulated in the CPEB3^−/−^ MII oocytes (CNOT7, ZSCAN4, CBFA2T3) and in the resulting 2cell embryos (SPI-C). We observed that mRNA expression correlates positively with protein expression (Figure 6A,B).

CPEBs are also involved in the polyadenylation of mRNAs and their translation CI; thus, we performed a poly(A) tail length assay (PAT) in the CPEB3^+/+^ and CPEB3^−/−^ oocytes to investigate the difference in the polyA tail lengths. The polyadenylation of 3′UTR mRNA coding for Cyclin B1 (*Ccnb1*), which is not DE in the CPEB3^−/−^ oocyte, is equal between genotypes (Figure 6C). In contrast, selected candidate mRNA 3′UTRs were differentially polyadenylated (Figure 6C). In all the analyzed candidates, we found significant differences in the size of the polyadenylated tails (Figure 6C).

Our data show aberrant protein expression of differentially expressed mRNAs in the CPEB3^−/−^ oocytes during the meiotic progression to MII, which is linked to the difference in the polyA tail length of the 3′UTR termini.

### 3.6. Injection of CPEB3 Reduced the Expression of Candidate Proteins in cKO Oocytes to Wild-Type Levels

To experimentally substitute for absent endogenous CPEB3 protein in the CPEB3^−/−^ oocytes, we injected CPEB3 protein into the GV oocytes (Figure S4) and subsequently analyzed the expression of selected candidate proteins, ZSCAN4 and CBFA2T3, in the in vitro matured MII oocyte. We detected levels of candidate proteins that were similar to those of the CPEB3^+/+^ MII oocytes after the injection of CPEB3 protein in the CPEB3^−/−^ oocyte (Figure 6D,E). However, our results showed that CPEB3 protein injection into CPEB3^−/−^ GV oocyte successfully rescued the protein expression of two selected candidates (ZSCAN4, CBFA2T3).

### 3.7. CPEB3 Regulates Translation of Specific mRNAs via 3′UTR

**Figure 7:**
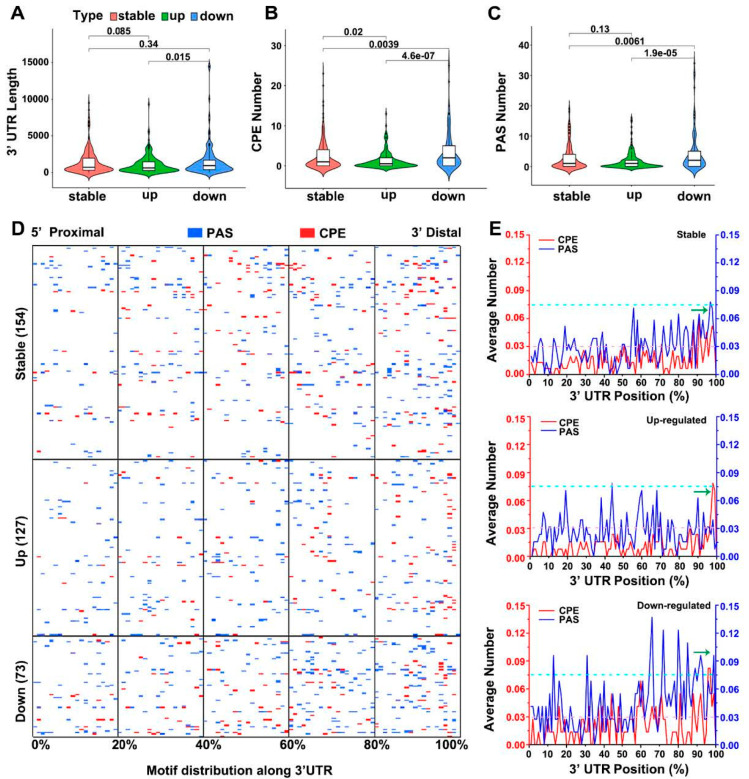
CPEB3 regulates translation of specific mRNAs via 3′UTR. Computational analysis of differentially expressed transcripts in CPEB3-depleted oocytes. (**A**) Analysis of 3′UTR length. (**B**) Number of cytoplasmic polyadenylation element (CPE) motifs. (**C**) Number of polyadenylation signal (PAS) motifs. Data are represented in File S1; stat values according to Wilcoxon test. (**D**) CPE (red) and PAS (blue) motif distribution in differentially expressed mRNAs in CPEB3-depleted MII oocytes. (**E**) CPE (red) and PAS (blue) motif distribution in differentially expressed subclass of transcripts in CPEB3-depleted MII oocytes.

The bioinformatic analysis of the DE transcripts in the CPEB3^−/−^ oocytes addressed their 3′UTR composition. The 3′UTR length, density, and spatial distribution of the polyadenylation signal (PAS) and cytoplasmic polyadenylation element (CPE) motives were investigated. The downregulated mRNAs had significantly longer 3′UTRs compared to the stable or upregulated transcripts (Figure 7A). The quantification of CPEs and PAS motifs showed a significant enrichment of PAS and CPEs in the downregulated mRNAs (Figure 7B,C), but the number of CPEs and PAS was equal to that of the stable mRNAs (Figure 7B,C). The spatial distribution of the CPE and PAS motifs showed a more distal localization of downregulated transcripts than with the stable or upregulated mRNAs (Figure 7D,E).

Our results show that CPEB3 regulates the fate of mRNAs containing long 3’UTRs, with CPE and PAS domains in the distant part of the mRNA transcripts.

## 4. Discussion

The RNA-binding protein CPEB3 mediates the fate of several identified mRNA targets [49,50]. Although the highest expression of CPEB3 has been found in the oocyte and zygote [41,42], the specific role of this maternal factor has not yet been elucidated. To gain insight into the function of CPEB3 in oocyte and early embryo development, we performed oocyte-specific downregulation of CPEB3 to characterize its importance in female reproduction.

Despite the presence of approximately 20% CPEB3 protein in the cKO oocytes, we observed a significant decrease in litter size. The remaining presence of the protein was likely due to its synthesis in the growing oocyte prior to ZP3-Cre activation, indicating a high stability of CPEB3 during the oocyte life cycle that was likely caused by its prion nature [51,52,53]. We hypothesize that the complete absence of CPEB3 in the oocyte would lead to female sterility due to impaired developmental competence of the oocyte. Since a 2cell arrest of more than 50% and significantly reduced transcription was observed in embryos derived from CPEB3^−/−^ oocytes, we hypothesize that the failure of ZGA is a major factor for subfertility. In addition, we found greatly reduced mRNA and protein levels of the transcription factor SPI-C (the upstream regulator of EiF1a and Oct4), whose accumulation prior to ZGA is essential for preimplantation development [54,55].

In contrast to the study by Fang et al. [40], we did not find any impairment of meiosis or follicle development. The authors conclude that subfertility is due to reduced folliculogenesis, which is associated with reduced GDF9 expression. We attribute the discrepancy between the results to differences in CPEB3 depletion strategies. The authors used a whole-body knock-out strategy [40], which may have had indirect secondary effects on oocytes through a somatic germline niche [38,39].

In *Xenopus* oocytes, CPEB4 takes over the role of CPEB1 during the second translational shift between MI and MII [36]. However, in mouse oocytes, deletion of CPEB4 has no effect on fertility, suggesting that there must be a different mechanism in mammals [31,37]. Our results show that CPEB3 contributes to developmental competence by controlling the stability of specific maternal transcripts, as revealed by an RNA-seq analysis involving a total of 355 genes of mouse MII oocytes [56]. Together with the slightly reduced translation observed in cKO-MII oocytes, it appears that CPEB3 replaces the role of CPEB1 for a specific subclass of mRNAs post nuclear envelope breakdown. Considering that the most highly represented category of differentially expressed genes (DEGs) are involved in transcriptional regulation, we hypothesize that destabilization of maternally derived transcriptional activators contributes to ZGA failure.

While the role of CPEB 1, 2, and 4 in cytoplasmic polyadenylation (CPA) in the oocyte is well documented [31,33,36,57], the molecular role of CPEB3 has been studied in other cell types, but its role remains unclear. It has been shown that CPEB3-mediated activation of GluA1/2 in the hippocampus does not require interaction with CPSF or a PAS, and CPEB3 has limited affinity for CPE [50]; however, the PAT assays of these genes showed poly-A tail elongation following CPEB3 activation [52,58]. By combining qRT-PCR, Western blot and poly-A tail length analyses, we demonstrated that downregulation of CPEB3 affects a subclass of RNAs where poly-A tail elongation and translation into MII is negatively affected. However, it is important to emphasize the limitations of our study. Our results show only an indirect relationship between CPEB3 and CPA because mechanistic studies on the physical interactions between CPEB3 and candidate RNAs are lacking. Interestingly, when comparing DEGs from two RIP-seq datasets of mouse neurons, some genes, including three common genes (*Stt3a*, *Sypl*, *Cnot7*), overlapped in the analyzed datasets [49,59], suggesting that similar interactions may exist in oocytes. Further studies are therefore required.

The analysis of gene expression in MII oocytes and 2cell embryos presented here also shows that the absence of CPEB3 leads to the stabilization of specific maternal mRNAs (of which 22% remained upregulated in the 2cell). The WB and poly-A tailing assay also confirmed impaired deadenylation and protein overexpression of two candidates in the MII oocyte (ZSCAN4, CBFA2T3). Importantly, ZSCAN4D is a 2cell-specific transcription factor [60] that is also transiently expressed in growing oocytes [61] and, if not fully knocked down in MII, leads to a loss of developmental competence [62]. The opposite DE pattern observed between the MII and 2cell stages could be attributed to impaired embryonic transcription, which could mask maternal mRNA overexpression. Another transcription factor, CBFA2T3 (MTG8), which recruits a large number of corepressors and histone-modifying enzymes [63], is stabilized in both the CPEB3^−/−^ oocyte and the embryo. Interestingly, one of the genes that was most highly overexpressed at both developmental stages was a replication-dependent histone, of which *Hist1h1e*, encoding H1.4, was one of the candidate genes validated by qPCR. H1.4 is associated with compaction of chromatin structure [64], leading us to speculate that its premature incorporation instead of the oocyte linker variant (H1FOO) might prevent proper chromatin loosening [65] in the pronuclei and sterically block the binding sites for TFs during the oocyte-to-embryo transition. Moreover, unbalanced histone stoichiometry could significantly affect ZGA, as reported in *Drosophilla* embryos [66]. Considering that somatic histones usually have a stem loop instead of a polyA tail at the 3′-UTR, the function of CPE found in the 3’-UTR of H1.4 and the possible switch to polyadenylation, as reported in other terminally differentiated tissues, refs. [67,68] remains unexplained.

Precise and selective deadenylation followed by degradation of maternal transcripts during M and Z decay is one of the prerequisites for successful transition from oocyte to embryo [69,70]. The mRNA Cnot7, which encodes a subunit of the CCR4-NOT deadenylase complex (the major deadenylase in oocytes), is recruited for translation by CPA during maturation [70]. Interestingly, downregulation of CNOT7 and impairment of its poly-A tail elongation was observed in CPEB3^−/−^ MII oocytes, suggesting a possible dual role of CPEB3 in deadenylation: (I) By repressing and subsequently activating Cnot7 via polyadenylation, which prevents the degradation of upregulated DEGs (such as Zscan4d) and/or (II) by deadenylating its own targets through interaction with BTG/TOB proteins, as reported in somatic cells [71], where CPEB3-TOB1 interaction recruited the deadenylation machinery. It would be interesting to investigate TOB1 as a potential maternal factor that recruits CCR-4-NOT to specific mRNAs (in contrast to BTG4 and ZFP36L targets). In addition, we cannot exclude the possibility that CPEB3-TOB1-mediated deadenylation activity is involved in the transient repression of dormant transcripts for future polyadenylation, perhaps through interactions with a deadenylase other than CCR-NOT (in a similar manner to the way that non-phosphorylated CPEB1 attracts PARN). Interestingly, in the available CPEB3 RIP-seq datasets from somatic cells [49,59], 17 and 16 genes among the upregulated 196 DEGs were identified as CPEB3 targets, respectively.

Recent research proposed *Cnot7* mRNA as a target of PAP-α, a nuclear poly-A polymerase that shuttles between the nucleus and cytoplasm and mediates CPA independently of CPE motifs [72]. Considering that CPEB3 and CPEB4 appear to have affinity for other U-rich elements [50], it is possible that the presumed “direct CPA” results from their interaction with PAP-alpha, but further research is needed.

As previously reported, different tissues show a global tendency to favor certain mRNA isoforms [73]. For example, neuronal tissues prefer isoforms that utilize distal PASs in 3′ UTRs, whereas the use of proximal PASs is more pronounced in blood cells and testicular tissue [73,74]. It appears that CPEB3 tends to favor the polyadenylation of distal PASs of mRNAs that are translationally quiescent at the onset of oocyte meiosis. This is consistent with a study by Zhang et al. (2021) [75], which showed that CPEB3 affects the fate of mRNA in lung cancer cells by influencing PAS choice and that its depletion leads to truncation of the 3′UTR, allowing transcripts to escape miRNA degradation [75]. Alternatively, another hypothesis should be considered: that deletion of CPEB3 in the short time window between ZP3-Cre activation and transcriptional silencing affects the intrinsic properties of 3′UTRs during pre-miRNA processing. This could result in these isoforms not being recognized by RBPs and their recruitment or degradation being impaired.

Interestingly, changes in 3′UTR length have recently been reported in human oocytes coupled with nuclear pre-mRNA processing [76]. First, deadenylated transcripts are partially degraded during maturation, followed by re-polyadenylation of truncated 3′UTRs by an unknown mechanism. The longer 3′UTR (with distal PAS) may be beneficial in the context of partial degradation, as its shortening affects the coding sequence. Our results show that the polysomal occupancy and protein content of CPEB3 are highest at the MII stage, decrease in zygotes, and disappear at the 2cell stage. The number of partially degraded intermediates (PDIs) increases significantly in zygotes [76]. Treatment with 3′-deoxyadenosine leads to a decrease in PDIs and inhibits polyadenylation and embryonic development, indicating the essential role of CPA after fertilization [77]. It would be interesting to investigate whether CPEB3 accumulation in MII is important not only for late meiotic translation but also for the re-polyadenylation of PDIs in mammals.

In summary, our results show that CPEB3 is dispensable for meiotic progression but essential for the maintenance of developmental competence in mouse oocytes. It enables sustained development to the blastocyst stage by shaping the maternal transcriptome and consequently the proteome, which is essential for oocyte-to-embryo transition and the transcriptional activation of the newly formed embryonic genome at the 2cell stage.

## Data Availability

The data included in this article are available in the article and in its online supplementary material. All data are available from the authors upon reasonable request. RNA-seq data were deposited in the GEO repository https://www.ncbi.nlm.nih.gov/geo/query/acc.cgi?acc=GSE239545 (accessed on 28 July 2023).

## Supplementary Materials

The following supporting information can be downloaded at: https://www.mdpi.com/article/10.3390/cells13100850/s1. Figure S1: Cpeb3 mRNA expression and phenotype analysis related to absence of CPEB3. (A) PCR analysis of Cpeb3 mRNA in oocytes. (B) Representative image of morphology of ovaries from specific genotypes. Scale bar, 1 mm. (C) Quantification of reproductive fitness of specific genotypes. Data are represented as the mean ± SEM from at least 11 breeding couples; ns, non-significant according to one-way ANOVA; Figure S2: Timing of first embryonic division is equal between genotypes. (A) Quantification of aberrant zygotes in relation to genotype. Data are represented as the mean ± SEM from at least eleven independent experiments; ns, non-significant, according to one-way ANOVA. (B) Quantification of timing of embryo cleavage to 2cell stage. From three biological replicates with depicted numbers of embryos; Figure S3: Principle component analysis of transcriptomes in MII oocytes and early 2cell embryo; Figure S4: Expression of microinjected CPEB3 protein in CPEB3-deficient oocytes. (A) Representative Immunoblot analysis of CPEB3-/- MII oocytes microinjected with exogenous CPEB3 protein (CPEB3+) in GV stage. CPEB3+/+ oocytes were used as a control. (B) Quantification of CPEB3 protein from (A). Data are represented as the mean ± SEM from three independent experiments; *** *p* < 0.001, according to one-way ANOVA, the expression of CPEB3 protein in the buffer-injected CPEB3+/+ oocytes (Control) from cKO females was set as 100%; Supplementary File S1: Gene ontology analysis showed that DE mRNAs coding mostly for genes involved in RNA expression, translation and transcription (Figure 5B and Supplementary File S1) in both oocytes and embryos. Supplementary Table S1: Primers and antibodies used in the study.
