# Supplementary material for: CPEB3 Maintains Developmental Competence of the Oocyte
Source: Cells. 2024 May 16;13(10):850. doi: 10.3390/cells13100850 (PMC11119423; doi:10.3390/cells13100850)
Supplement: Supplementary file 1 [file cells-13-00850-s001.zip › cells-2980582 Supplementary Figures.pdf]

Fig. S1

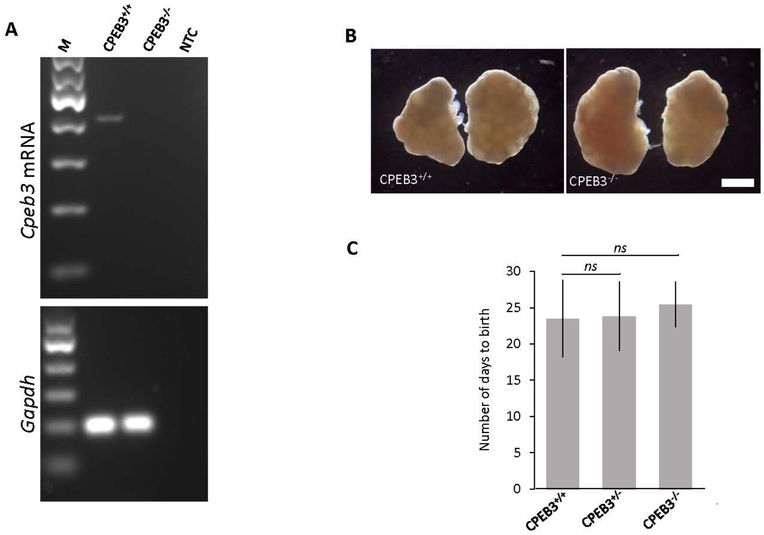

Fig. S1: *Cpeb3* mRNA expression and phenotype analysis related to absence of CPEB3. A) PCR analysis of *Cpeb3* mRNA in oocytes. **B**) Representative image of morphology of ovaries from specific genotypes. Scale bar, 1mm. **C**) Quantification of reproductive fitness of specific genotypes. Data are represented as the mean $\pm$ SEM from at least 11 breeding couples; *ns*, non-significant according to one-way ANOVA.

Fig. S2

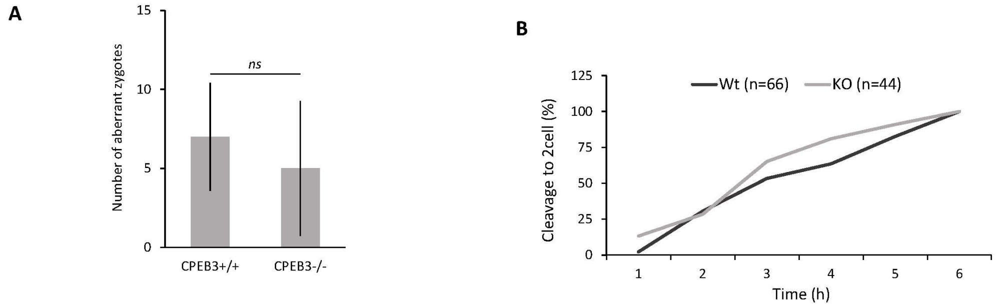

Fig. S2: Timing of first embryonic division is equal between genotypes. **A**) Quantification of aberrant zygotes in relation to genotype. Data are represented as the mean $\pm$ SEM from at least eleven independent experiments; *ns*, non-significant, according to one-way ANOVA. **B**) Quantification of timing of embryo cleavage to 2cell stage. From three biological replicates with depicted numbers of embryos.

Fig. S3

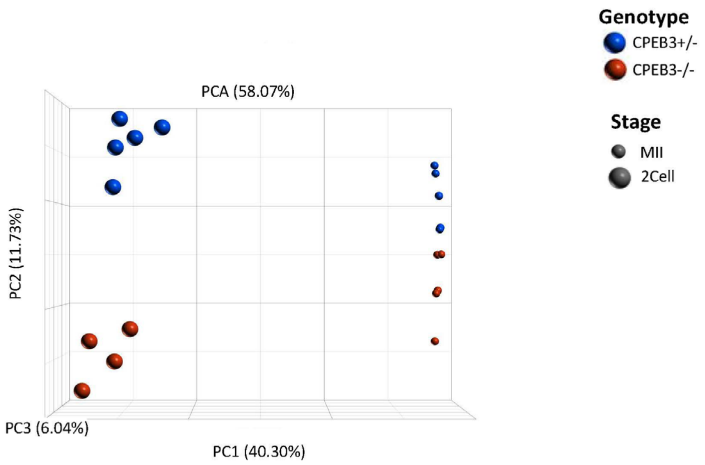

Fig. S3. Principle component analysis of transcriptomes in MII oocytes and early 2cell embryo.

Fig. S4

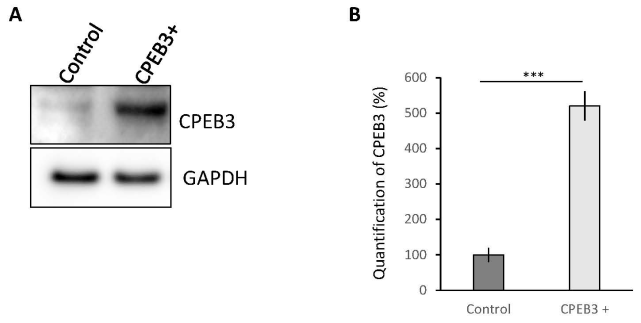

Fig. S4: Expression of microinjected CPEB3 protein in CPEB3-deficient oocytes. A) Representative Immunoblot analysis of CPEB3$^{-/-}$ MII oocytes microinjected with exogenous CPEB3 protein (CPEB3+) in GV stage. CPEB3$^{+/+}$ oocytes were used as a control. **B**) Quantification of CPEB3 protein from **A**). Data are represented as the mean ± SEM from three independent experiments; *** $p < 0.001$, according to one-way ANOVA, the expression of CPEB3 protein in the buffer-injected CPEB3$^{+/+}$ oocytes (Control) from cKO females was set as 100%.

Supplementary Table 1: **Primers and antibodies used in the study.**

Primers used for qRT-PCR

Official symbol	Forward 5´- 3´	Reverse 5´- 3´	Gene Bank ID	Amplicon size (bp)
Cnot7	GCAGGATCTGACTCACTGCTTA	*GTCATGACTGCTTGCTGGCT*	NM_011135.5	181
Zscan4d	ACTTTTATGTTTTGTTTTTTATTGTG	CGAGTCTCTGACCGCCGAACCCA	NM_001100186.1	134
Cbfa2t3	CCGACCGTGAGGAACTCAAC	CATGTAGCCAGACAGGGTCC	NM_009824.2	170
Obox5	TCATGTTGGAGCCTTGGTTCTC	TTTGGATGCAAGGAGGGACC	NM_145709.2	90
Spi-C	TGGAATGTCACCCACAGAGA	CTGTACGGATTGGTGGAAGC	NM_011461.3	97
Hist1h1e	TCTCTCCTCACACGCTTCGC	GGGCCTTCTTCTTGACGGGT	NM_015787.4	94
Eif1a	AACAGGCGCAGAGGTAAAAA	CTTATATGGCACAGCCTCCT	NM_001374654.1	156
Zfp770	ACAGCACCACGAAGAGAAGA	TGGGTTGACTGTCGGAAAGA	NM_175466.5	157
Gapdh	TGGAGAAACCTGCCAAGTATG	GGTCCTCAGTGTAGCCCAAG	XM_001476707.3	92

Primers used for PAT-assay

Official symbol	Forward 5´- 3´	Reverse 5´- 3´	Gene Bank ID
Anchor primer	-	GCGAGCTCCGCTTTTTTTTTTTT	-
Cnot7	GACCAGAGCATGTGTTGGAAGATTTTG	-	NM_011135.5
Zscan4d	CGAGTCTCTGACCGCCGAACCCA	-	NM_001100186.1
Cbfa2t3	CATCTATCTGGGTGCTGGGT	-	NM_009824.2
Obox5	AGTGCGATTGTTTTAAAGTC	-	NM_145709.2
Zfp770	CATGTTAAATGATGGTTTGC	-	NM_175466.5
Ccnb1	CAAGTGCATTCTCTCAGTGCCCTCCACAGTGT	-	NM_172301.3

Antibodies used for WB

Name (cat.#)	Company
CPEB3 (SAB2100473)	SigmaAldrich
ZSCAN4 (ab97748)	Abcam
SPI-C (PA5-67537)	Invitrogen
CBFA2T3 (17190-1-AP)	Protein tech
H1.4 (bs-10334)	Bioss
CNOT7 (WH0029883M1)	SigmaAldrich
GAPDH (97166)	Cell Signalling Tech.
GAPDH (G9545)	SigmaAldrich

Supplementary File 1: Gene ontology analysis showed that DE mRNAs coding mostly for genes involved in RNA expression, translation and transcription (**Fig. 5B and Supplementary File 1**) in both oocytes and embryos.
